# Energy metabolism during exercise in patients with β‐enolase deficiency (GSDXIII)

**DOI:** 10.1002/jmd2.12232

**Published:** 2021-06-14

**Authors:** Astrid Emilie Buch, Olimpia Musumeci, Ralph Wigley, Mads Peter Godtfeldt Stemmerik, Anne‐Sofie Vibæk Eisum, Karen Lindhardt Madsen, Nicolai Preisler, David Hilton‐Jones, Ros Quinlivan, Antonio Toscano, John Vissing

**Affiliations:** ^1^ Copenhagen Neuromuscular Center, Rigshospitalet, University of Copenhagen Copenhagen Denmark; ^2^ Neurology and Neuromuscular Disorders Unit, Department of Clinical and Experimental Medicine University of Messina Messina Italy; ^3^ Enzyme Laboratory, Department of Chemical Pathology Cameilia Botnar Laboratories, Great Ormond Street Hospital for Sick Children London UK; ^4^ Department of Clinical Neurology West Wing, John Radcliffe Hospital Oxford UK; ^5^ Dubowitz Neuromuscular Centre, Great Ormond Street Hospital for Children NHS Foundation Trust London UK

**Keywords:** β‐enolase deficiency, exercise intolerance, exercise metabolism, GSDXIII, maximal exercise capacity, metabolic myopathy

## Abstract

**Aim:**

To investigate the in vivo skeletal muscle metabolism in patients with β‐enolase deficiency (GSDXIII) during exercise, and the effect of glucose infusion.

**Methods:**

Three patients with GSDXIII and 10 healthy controls performed a nonischemic handgrip test as well as an incremental cycle ergometer test measuring maximal oxidative consumption (VO_2max_) and a 1‐hour submaximal cycle test at an intensity of 65% to 75% of VO_2max_. The patients repeated the submaximal exercise after 2 days, where they received a 10% iv‐glucose supplementation.

**Results:**

Patients had lower VO_2max_ than healthy controls, and two of three patients had to stop prematurely during the intended 1‐hour submaximal exercise test. During nonischemic forearm test, all patients were able to produce lactate in normal amounts. Glucose infusion had no effect on patients' exercise capacity.

**Conclusions:**

Patients with GSDXIII experience exercise intolerance and episodes of myoglobinuria, even to the point of needing renal dialysis, but still retain an almost normal anaerobic metabolic response to submaximal intensity exercise. In accordance with this, glucose supplementation did not improve exercise capacity. The findings show that GSDXIII, although causing episodic rhabdomyolysis, is one of the mildest metabolic myopathies affecting glycolysis.

**Synopsis**

Patients with GSDXIII suffer from a mild glycolytic defect that limits maximal oxidative capacity.

## 1 INTRODUCTION

Enolase is the enzyme responsible for the penultimate step in glycolysis as it catalyzes the conversion of 2‐phosphoglycerate to phosphoenolpyruvate. The enzyme exists in three tissue‐specific isoforms in adults, each composed of a homodimer of the α‐, β‐, or γ‐subunit of enolase.^1^ ENO3, a homodimer of the subunit β‐enolase, is responsible for nearly all enolase activity in skeletal muscle tissue.^2^

Recessive mutations in the autosomal ENO3 gene (*ENO3*) have been reported to cause β‐enolase deficiency, also called glycogen storage disease number XIII (GSDXIII). GSDXIII is extremely rare with only five patients reported worldwide so far.^3^, ^4^, ^5^ These patients all have a history of exercise intolerance and episodic rhabdomyolysis without cardiac involvement. So, clinically these patients resemble other glycogenosis with episodic symptoms related to energy deficiency during exercise,^6^, ^7^ but exercise capacity and metabolism during exercise has never been studied in GSDXIII‐patients. In this study, we had the opportunity to examine three of the five known patients with GSDXIII.

Because of the relative block in glycolysis, we hypothesized that the GSDXIII‐patients, like other partial defects of glycolysis,^7^, ^8^ retain a partial glycolytic flux, limiting only high‐intensity exercise capacity. We also hypothesized that iv‐glucose supplementation will not improve exercise capacity in GSDXIII‐patients unlike that seen in a number of more proximal defects of glycolysis and glycogenolysis.^6^, ^9^, ^10^, ^11^, ^12^, ^13^, ^14^, ^15^

## 2 MATERIALS AND METHODS

### 2.1 Participants

We investigated three unrelated men with GSDXIII. The diagnoses were verified genetically and biochemically (Table 1).^3^, ^4^, ^5^ The patients all experienced exercise intolerance, contractures, and repeated episodes of rhabdomyolysis. Two of the three have prolonged episodes of myalgia with exercise (Table 1). The patients had no muscle weakness or atrophy. The participants did not take any medication. Physical activity level in the three patients varied: patient 1 did not exercise regularly, patient 2 performed moderate‐intensity exercise for 5 hours twice a week and walked approximately 1 hour per day, and patient 3 walked 1 hour almost daily. Cycle ergometer tests results were compared to exercise tests performed by 10 healthy, sedentary controls (average age 35 ± 11 years, weight 70 ± 11 kg, average maximal oxygen consumption 46 ± 7 mL O_2_ min^−1^), who cycled at the same absolute workload as the patients. Results from the nonischemic handgrip test were compared to results from nine other healthy controls (average age 34 ± 3 years, weight 77 ± 5 kg, maximal voluntary contraction [MVC] 45 ± 4 kg). Both groups of healthy controls were recruited for this and other metabolic studies that have been reported earlier.^12^, ^16^, ^17^

**TABLE 1:** Clinical, genetic, and biochemical characteristics, and results from maximal exercise test in patients and control group.

	Patient 1	Patient 2	Patient 3	Healthy controls (N = 10)	
				Mean ± SD	Range
*Baseline demographics*					
Age (y)	50	21	41	33 ± 11	19‐56
Body Mass Index (kg m^−2^)	35	29	25	22 ± 3	19‐27
Mutation, homozygous	c.452A>G	c.1070G>A	c.559G>A	NA	NA
Enzyme activity (% of normal)	20^a^	33^b^	10^a^	NA	NA
Onset of exercise intolerance (y)	20	15	0	NA	NA
Myalgia	Yes	Yes	No	NA	NA
Episodic rhabdomyolysis	Yes	Yes	Yes	NA	NA
Peak creatine kinase (U L^−1^)	200 000	193 000	75 000	NA	NA
*Maxtest*					
VO_2max_ (mL kg^−1^ min^−1^)	12*	22*	28*	46 ± 6	36‐56
Heart rate_max_ (bpm)	126	188	174	191 ± 9	181‐210
Workload_max_ (J s^−1^)	35	175	130	237 ± 82	160‐390
Lactate_max_ (mmol L^−1^)	1.2	8.6	6.7	11.5 ± 2.8	9‐17.3
Creatine kinase_rest_ (U L^−1^)	126	931	94	NA	NA
Creatine kinase_peak_ (U L^−1^)	374	2530	121	NA	NA

NA assigned when value is Not Applicable.

^*^ Result significantly different from mean in healthy control group, *p* < 0.05.

^a^ As reported in Musumeci et al, *Journal of Neurology*, 2014.^3^

^b^ As reported in Wigley et al, *JIMD Reports*, 2019.^5^

### 2.2 Nonischemic forearm exercise test

Each participant performed a nonischemic forearm exercise test by squeezing a handheld dynamometer at MVC every other second for a full minute with their dominant hand.^16^ The best of three attempts at MVC was determined before the test, and force was monitored every 10 seconds throughout the test. Venous lactate and ammonia were measured before (baseline), at the end of exercise, and 1 and 5 minutes postexercise. Blood samples were drawn from a venous catheter in the median cubital vein of the exercising arm.

### 2.3 Maximal exercise

Each participant performed an incremental cycle test to exhaustion on an electronically braked cycle ergometer (Lode Excalibur, The Netherlands) to determine maximal oxygen consumption (VO_2max_), maximal heart rate (HR), and maximal workload (*W*_max_).

### 2.4 Submaximal exercise

The next day, patients and healthy controls performed an intended 1‐hour cycling bout at a workload corresponding to 65% to 75% of the patient's VO_2max_. The participants were fasted overnight. Breath‐by‐breath gas exchange was measured with a metabolic cart (Cosmed, Italy). Blood samples were drawn prior to the exercise and every 10 minutes during exercise. Concentrations of lactate, free fatty acids (FFAs), and glucose were measured.

After a day of rest, the GSDXIII‐patients repeated the submaximal exercise test, this time with a 10% iv‐glucose solution. A bolus of 10% iv‐glucose was delivered 10 minutes before the beginning of the exercise, followed by a constant infusion of 4.7 mL kg^−1^ min^−1^. The effect of iv‐glucose was evaluated by duration of exercise, HR, and rate of perceived exertion (RPE).^18^

### 2.5 Calculations

Whole‐body fatty acid oxidation (FAO) was calculated by indirect calorimetry using a nonprotein respiratory quotient.^19^ Differences in mean exercise values and oxidation rates between patients and controls were assessed using an unpaired Student's *t* test, and with a paired *t* test when assessing the effect of iv‐glucose supplementation. A *P* value of <.05 (two‐tailed testing) was considered significant. Results are shown as mean ± SD.

## 3 RESULTS

### 3.1 Nonischemic forearm exercise test

**FIGURE 1:**
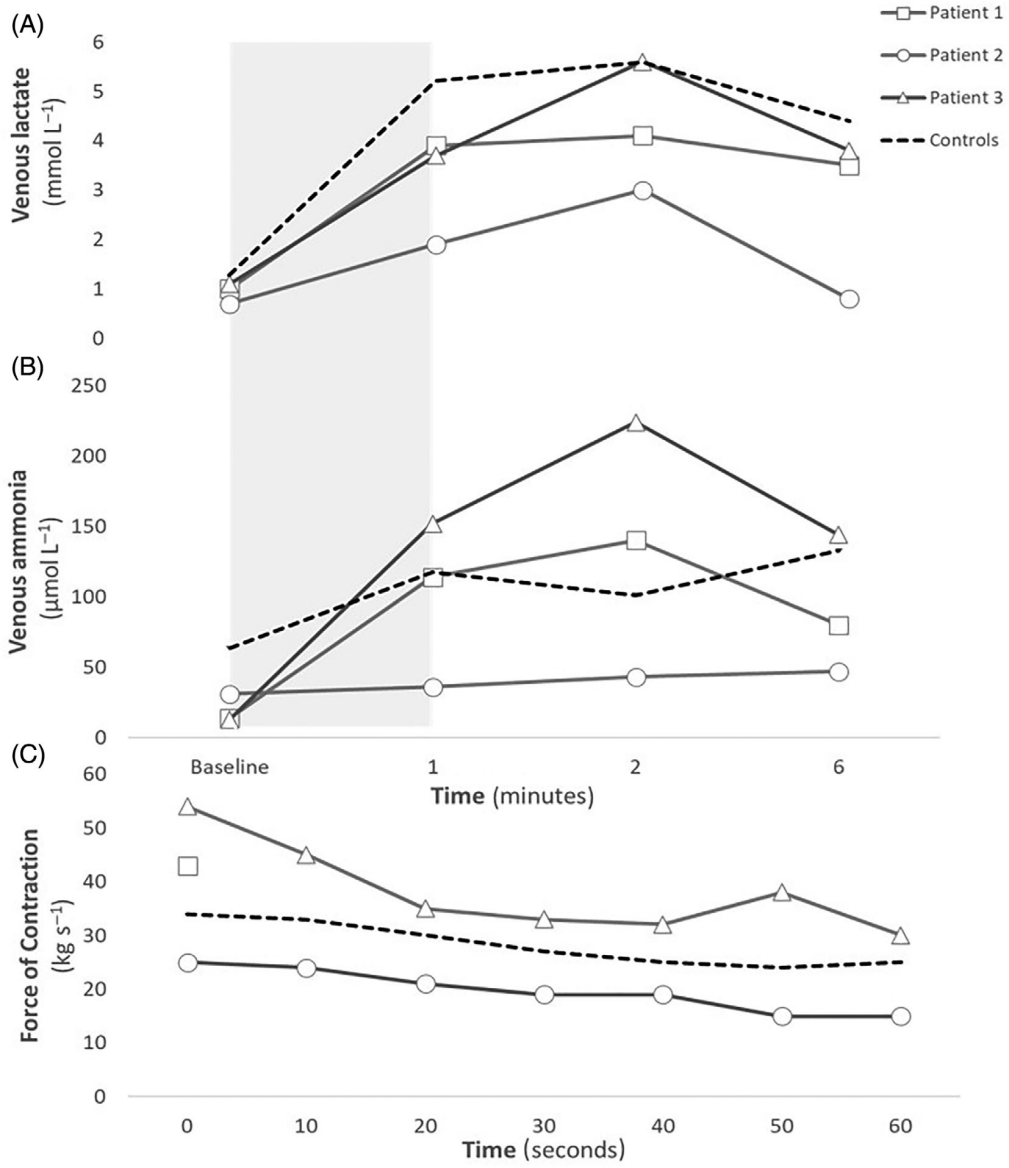
Nonischemic handgrip test in three patients with β‐enolase deficiency and nine healthy controls. Participants squeezed a handheld dynamometer with maximal force at a frequency of 1 Hz 60 seconds (gray area). Contraction force was documented every 10 seconds, except in patient 1. Average for healthy controls is shown by the dotted line

MVC, peak lactate, and ammonia levels in the patients were comparable to the values in the healthy controls (Figure 1) (peak p‐lactate 4.2 ± 2.1 mmol L^−1^ vs 5.6 ± 1.1 mmol L^−1^ in controls, *P* = .1, and peak p‐ammonia 137 ± 89 μmol L^−1^ vs 141 ± 33 μmol L^−1^ in controls, *P* = .9), except in patient 2 in whom both metabolites reached half the values of the healthy controls, indicating low effort during handgrip. Patient blood glucose levels were between 4 and 8 mmol L^−1^ and remained constant throughout the test (maximal variation 0.2 mmol L^−1^).

### 3.2 Maximal exercise

Average VO_2max_ and *W*_max_ in the patients was less than half that in the healthy controls (Table 1), though difference in *W*_max_ did not reach statistical significance (*P* = .02 and *P* = .07, respectively). Peak lactate was generally lower in patients than in controls, reflecting the lower external work. Patients 2 and 3 reached values of HR_max_ that were expected for their age, while patient 1 reached a HR_max_ 30% lower than expected for his age.^20^

### 3.3 Submaximal exercise

Only one patient completed 60 minutes of exercise in both tests, while the others stopped prematurely: patient 1 because of contractures, and patient 2 because of fatigue. HR was stable throughout exercise, without signs of a second wind phenomenon.^9^

**FIGURE 2:**
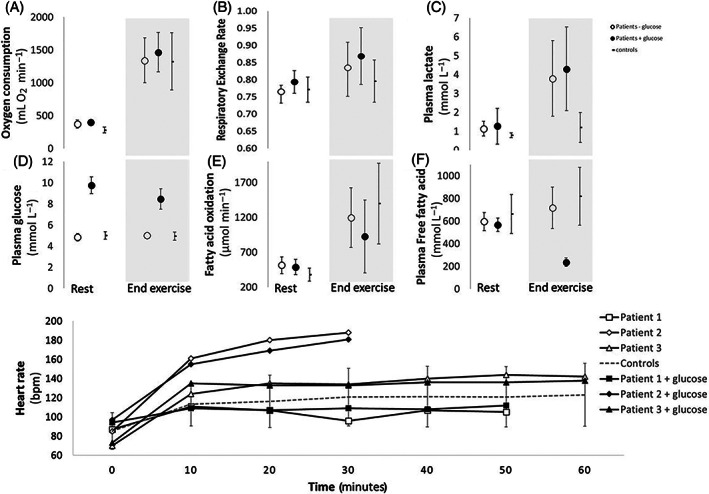
Metabolic parameters in three patients with GSDXIII with and without glucose supplementation and 10 healthy controls during submaximal exercise up to 60 minutes: A, Oxygen consumption rate; B, Respiratory exchange rate; C, Plasma lactate; D, Plasma glucose; E, Fatty acid oxidation; F, Plasma free fatty acids; G, Heart rate. Results illustrated as an average ± SD

The constant workload corresponded to a higher relative workload for the patients than for the healthy controls as reflected by a higher average VO_2_ (74% ± 10% vs 51% ± 15% of VO_2max_, *P* = .016), a higher average HR (87% ± 8% vs 70% ± 15% of HR_max_, *P* = .008) (Figure 2) as well as higher RPE at the end of exercise (18 ± 2 vs 13 ± 4, *P* = .011).

### 3.4 Metabolism during submaximal exercise

Glucose levels remained within normal range (4.1‐5.5 mmol L^−1^) during the test in the GSDXIII‐patients and healthy controls (Figure 2). Plasma lactate tripled in the GSDXIII‐patients compared to end lactate in the healthy controls and the respiratory exchange rate rose (0.83 ± 0.11 vs 0.81 ± 0.06 in controls, *P* = .054), indicating a significant contribution from carbohydrate oxidation to the exercise metabolism (Figure 2), a result of the higher relative workload in the patients favoring glycolytic metabolism.

Circulating FFAs and FAO both increased with exercise in patients and healthy controls.

### 3.5 Fuel supplementation

Duration of exercise, average HR and VO_2_, and rate of perceived exertion at the end of the exercise were not significantly affected by the iv‐glucose supplementation in the GSDXIII‐patients (Figure 2).

### 3.6 Safety

No patient experienced myoglobinuria. Plasma creatine kinase (p‐CK) tended to increase after exercise in all patients but not to levels indicating rhabdomyolysis (Table 1).

## 4 DISCUSSION

The main finding in this study is that patients with GSDXIII have a reduced oxidative capacity during exercise to about half that in healthy matched controls. Despite this reduced peak exercise capacity and repeated episodes of rhabdomyolysis in all patients, glycolytic flux was near normal at submaximal exercise intensities. The findings therefore suggest that this rare muscle glycogenosis is relatively mild, only demasking a limitation in the glycolysis during peak or higher intensity exercise, in line with what has been reported for other defects of distal glycolysis,^7^, ^8^, ^21^ which however on rare occasions can progress to kidney failure as observed earlier in one of our patients. Because GSDXIII is a rare glycolytic disorder, it may be unrecognized due to its mild nature and could be missed if not considered in the differential diagnosis of rhabdomyolysis. It is possible that a future patient with GSDXIII could present with a more severe clinical phenotype as a result of lower residual enzyme activity and a more significant glycolytic block than the patients we have in examined in this study. This is only speculative, however, as we have studied three out of the five known cases of β‐enolase deficiency worldwide who all show a relatively mild phenotype.

Iv‐glucose supplementation did not change exercise capacity, in contrast to what has been reported in patients with more proximal defects of glycolysis, such as myophosphorylase deficiency (McArdle disease) and phosphoglucomutase deficiency, showing that the increased glucose availability does not increase the glycolytic rate in patients with GSDXIII.^9^, ^21^ The lack of effect is in line with findings in other defects of glycolysis, such as phophofructokinase and phosphoglycerate mutase deficiencies.^7^, ^11^ On the contrary, oral ingestion of sucrose or other types of carbohydrate in patients with GSDXIII, where insulin response is much greater than with IV administered glucose supplementation, might actually be harmful. Oral sucrose supplementation would suppress FAO to an even higher degree than is seen in our study and the effect could be reminiscent of the “out‐of‐wind” phenomenon in phosphofructokinase deficiency.^11^

Our results indicate that residual β‐enolase activity as low as 10% supports normal lactate production. Normal lactate production has also been found in other partial defects of glycolysis such as phosphoglycerate mutase deficiency and patients with less severe phosphoglucomutase type 1 deficiency.^7^, ^21^ This is contrary to patients with other glycogen storage diseases, such as McArdle disease, phosphofructokinase and phosphoglycerate kinase deficiencies, and more severe cases of phosphoglucomutase type 1 deficiency, who clinically present with more severe phenotypes and lower glycolytic capacity.^6^, ^8^, ^10^, ^22^ Residual enzyme activity levels and kinetic profiles of enolase in the GSDXIII patients we studied have been reported earlier,^3^, ^5^ but are not directly comparable. Therefore, any correlation between residual activity and lactate production cannot be determined.

Despite all being able to produce lactate as a response to high intensity exercise, two of the three GSDXIII patients were not able to do this consistently throughout the different exercise protocols. This finding suggests a level of underperformance. All three patients had been cautioned against exercising by treating health care professionals. These warnings, combined with personal histories of episodic rhabdomyolysis and unfamiliarity with strenuous exercise, could explain the unintentional underperformance, and might also be the reason that an earlier study has indicated that these patients have an impaired ability to produce lactate.^4^ In this study, one patient had increased CK levels at rest, and also had a significant rise in CK after cycle testing, without any clinical symptoms. No adverse event occurred in response to the exercise tests. The findings of this study suggest that submaximal exercise could be a beneficial exercise form for this type of patients. However, further research into the safety of exercise training is necessary and important to possibly prevent a detrimental sedentary lifestyle in these patients.

## 5 CONCLUSION

Like other milder glycogen storage diseases, patients with GSDXIII have reduced maximal oxidative capacity, but sufficient flux through the glycolysis to support lactate production during submaximal exercise intensity.

## CONFLICT OF INTEREST

The authors declare no potential conflict of interest.

## AUTHOR CONTRIBUTIONS

Astrid Emilie Buch designed the study, collected and analyzed the data, and wrote the manuscript. John Vissing, Nicolai Preisler, and Karen Lindhardt Madsen designed the study and collected the data. Mads Peter Godtfeldt Stemmerik and Anne‐Sofie Vibæk Eisum collected the data. All authors reviewed and edited the manuscript, contributed to discussion, and approved the final manuscript.

## INFORMED CONSENT

All procedures followed were in accordance with the ethical standards of the responsible committee on human experimentation (institutional and national) and with the Helsinki Declaration of 1975, as revised in 2000. The study was approved by The Committee on Health Research Ethics of the Capital Region of Denmark (H‐15015150). Informed consent was obtained from all patients for being included in the study.
